# EFFECT OF CHRONIC INGESTION OF WINE ON THE GLYCEMIC, LIPID AND BODY WEIGHT HOMEOSTASIS IN MICE

**DOI:** 10.1590/0102-6720201600030005

**Published:** 2016

**Authors:** Sebastião Barreto de BRITO-FILHO, Egberto Gaspar de MOURA, Orlando José dos SANTOS, Euler Nicolau SAUAIA-FILHO, Elias AMORIM, Ewaldo Eder Carvalho SANTANA, Allan Kardec Dualibe BARROS-FILHO, Rennan Abud Pinheiro SANTOS

**Affiliations:** 1 Academic League in Experimental Surgery Laboratory, School of Medicine, Federal University of Maranhão and State University of Rio de Janeiro, Rio de Janeiro, RJ, Brazil

**Keywords:** Homeostasis, Mice, Wine

## Abstract

**Background:**

The health benefits associated with moderate wine consumption, as with ethanol and phenolic compounds, include different mechanisms still little understandable.

**Aim:**

Evaluate glycemic and weight variations, and the deposit of triglycerides, cholesterol and liver glycogen with red wine consumption.

**Methods:**

60 ApoE knockout mice were divided into three groups of 20: Wine Group (WG), Ethanol Group (EG) and Water Group (WAG). They received daily: WG 50 ml of wine and 50 ml water; EG 6 ml ethanol and WAG 94 ml of water. All groups were followed for four months. The food intake was monitored daily, in the period from eight to ten hours and held every five days. The measurement of water intake was also made every five days. The weighing of the animals took place every ten days.

**Results:**

The WG had higher weight increase as compared to the other groups. The concentration of hepatic triglyceride was higher in WG (57%) and the EG group was lower (31.6%, p<0.01) than the control. The concentration of cholesterol was lower in the WG (23.6%), as well as EG (24.5%, p<0.05). The concentration of glycogen was higher in WG (16%) and fasting blood glucose was higher in EG compared to the other groups but not both demonstrated a statistically significant difference.

**Conclusion:**

The WG increased triglyceride and WAG decreased cholesterol. The triglyceride may be increased due to the high caloric value of wine or some unknown property that led to significant increase in subcutaneous andretroperitoneal fat in mice.

## RESUMO

**Racional:**

Os benefícios para a saúde associados ao consumo moderado de vinho, como etanol e compostos fenólicos, incluem mecanismos diferentes ainda pouco compreensíveis.

**Objetivo:**

Avaliar as variações da glicemia, peso e depósito de triglicrideos, colesterol e glicogênio hepático com o uso de vinho tinto.

**Métodos:**

Sessenta camundongos ApoE knockout foram divididos em três grupos de 20: Grupo do Vinho (WG), grupo do Etanol (EG) Grupo Água (WAG). Cada grupo recebeu diariamente: WG 50 ml de vinho e 50 ml de água; EG 6 ml de etanol e WAG 94 ml de água.

**Resultados:**

O WG teve aumento de peso mais elevado em comparação com os outros grupos. A concentração de triglicerídeos foi maior no WG (57%) e no grupo EG inferior (31,6%) do que no controle (p <0,01). A concentração de colesterol foi inferior no WG (23,6%) e no EG (24,5%, p<0,05). A concentração de glicogênio foi maior no WG (16%); a glicemia capilar foi maior no EG em comparação com os outros grupos, mas não demonstrou diferença estatisticamente significativa.

**Conclusão:**

Triglicerídeos ficaram aumentados no WG e o colesterol diminuiu no WAG. Os triglicerídeos podem ter aumentado devido ao alto valor calórico do vinho ou alguma propriedade desconhecida que levou ao aumento significativo da gordura subcutânea e retroperitoneal nos camundongos.

## INTRODUCTION

The first reports of wine consumption either as drink or medicinal purposes date back 7,000 years B.C. Registration on papyruses from Egypt and tablets of the Sumerians (about 2200 B.C.) brought healing recipes based on wine, enshrining it as the oldest documented prescription. Hippocrates (around 400 B.C.) used and recommended wine as disinfectant, a drug associated with other drugs and as part of a healthy diet^27^. Galeno (II A.D.) employed the wine in healing the wounds of the gladiators, as disinfectant^23^.

The use of wine was popularized throughout history for various purposes. However, from the late nineteenth century, the understanding of wine as medication began to change. Alcoholism has been considered disease and the harms and benefits of its consumption began to be studied. References for benefits with regular consumption emerged in 1992 with the publication of the "French Paradox". The term meaning an apparent incompatibility between the wasteful consumption of lipids in the diet with the low incidence of cardiovascular diseases which can be attributed to the regular consumption of red wine that has phenolic compounds in its composition - in particular flavonoids -, which inhibit the reaction of LDL oxidation^7^ ^,^ ^10^.

Several studies were held evaluating the benefit of alcoholic beverages, with emphasis on wine consumption or different alcoholic beverages (wine, beer and distillates) and the risk of death from cardiovascular diseases in a population of 13,000 people in Denmark in a year. It has been shown that the daily consumption of wine significantly reduced the risk of death by cardiovascular diseases, while other alcoholic beverages led to little or no change. Notwithstanding there are also reports that after a certain level of daily consumption, the wine carries the opposite effect, increasing the risk of death from cirrhosis and other diseases. It is clear that wine reduced the risk of vascular diseases by containing other compounds besides ethanol in the beverage^29^.

The benefits of wine consumption are related to ethanol and phenolic compounds, particularly resveratrol. The function of resveratrol in grape is to protect against fungi, bacteria, viruses and solar radiation, found in the grape bark, seed and pulp. The possible beneficial health effects are multiples but not very well understood^1^ ^,^ ^3^ ^,^ ^5^ ^,^ ^6^ ^,^ ^8^ ^,^ ^9^ ^,^ ^11^ ^,^ ^12^ ^,^ ^14^ ^,^ ^15^ ^,1617,^ ^29^ ^,^ ^30^.

As wine is widely used in diets and has a high caloric intake, its contribution to weight gain and glycemic control can be considerable. Thus, with regular consumption there is greater stock of lipids, body weight increase and consequently onset of obesity. The accumulation of fat takes place preferably in the abdomen. Ethanol is also considered an appetite enhancer operating in various neurochemical systems by inhibiting leptin and serotonin or increasing the effect of gamma-aminobutyric acid, opioids and neuropeptide Y^19^ ^,^ ^24^.

The aim of this study is to evaluate the chronic effect of red wine on the glycemic, lipid and body weight homeostasis in genetically modified ApoE knockout mice. 

## METHODS

### Animals and experimental groups 

Sixty adult male ApoE knockout mice were used, with average weight of 30 g. The animals were kept at temperature of 22±3°C with light/dark periods of 12 h. The experiment was conducted in accordance with the ethical principles for animal experimentation and was approved by the Ethics and Animal Experimentation Committee of the Agricultural Sciences Center of the Veterinary Medicine Course, State University of Maranhão, under the protocol 002/2011.

The animals were divided into three groups: Wine Group (WG), Ethanol Group (EG) and Water Group (WaG) being 20 ApoE knockout mice in each. They were kept in 15 cages with four animals, receiving standard feed and identified in each cage with markings in the head, upper tail, lower tail and unmarked, for four months.

In the WG, the animals received a commercial normal diet for the species with free access to water plus wine, 50 ml wine+50 ml water. The red wine was from pinot noir grapes (5.13 mg resveratrol/l 13% vol). In the EG, the animals underwent the same diet with free access to the water recipient containing 6 ml ethanol+94 ml water. WaG received the same diet of the above groups with free access only to water (100 ml).

The food intake was monitored daily, in the period from eight to ten hours and held every five days. The measurement of water intake was also made every five days. The weighing of the animals took place every ten days.

### Animal death 

All groups were followed for four months. The day before death, all were fasted and after a period of 12 h, fasting glucose was gauged with the aid of Accu-Chek ative^(r)^ glucometer, and then they were induced to death with lethal dose of anesthetics. The combination of ketamine (Ketalar^(r))^ at a dose of 15 mg/kg with xylazine (Rompum^(r))^ at a dose of 3 mg/kg was made intramuscularly. 

After the death was performed the opening of the abdominal cavity. The liver and visceral (retroperitoneal and epididymal) and subcutaneous (inguinal) fat was collected and weighed. Adipose tissue deposits were collected from both sides of the animal body and it was established as the retroperitoneal fat the deposition existing around each kidney and along the lumbar muscles, and the epididymal fat as the adipose tissue around both ureters, bladder and epididymis.

Part of the epipidimal adipose tissue was stored in Millonig formalin solution (1.27 mol/1 formaldehyde in 0.1 M phosphate buffer, pH 7.2) for fixation and subsequent histological processing for microscopy.

### Morphometry of adipose tissue

After 48 h fixation the material was taken for histological processing consisting of dehydration in increasing concentrations of alcohol, diaphanization in xylene and inclusion in Paraplast Plus (Sigma-Aldrich Co., St. Louis, MO, USA). Subsequently, it was obtained random and non-sequential cuts of 5 µm for the preparation of histological slides, which were then subjected to hematoxylin-eosin staining. Ten photomicrographs per animal were taken with Olympus DP71 camera and Olympus BX40 fluorescence microscope. Analyzes of the sectional area of adipocytes were performed using the program Image-Pro Plus version 5.0.

### Biochemical analysis 

Hepatic glycogen was evaluated through glucose produced by the hydrolysis of the hepatic glycogen using commercial kit and specific technique for it.

Hepatic triglyceride was analysed by liver samples (50 mg) 

homogenized and centrifuged. The triglyceride content was quantified by colorimetric commercial kit according to the manufacturer's specifications.

Hepatic cholesterol was measured from the same homogenate of the triglycerides processing with colorimetric analysis.

### Statistical analysis

Data were analyzed statistically using the Stata 10.0 software for Windows. Results were expressed as mean±standard deviation. To compare the means was used analysis of variance (ANOVA) and the T test for unpaired samples with equal variances. The level of significance to reject the null hypothesis was 0.05.

## RESULTS

### Body mass evaluation

**FIGURE 1:**
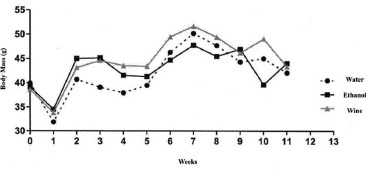
Evaluation of body mass in weeks

The amount of intake of food and liquids were similar in all groups. In groups comparison, WG body mass was approximately 6.35% higher than the other groups, showing a statistically significant difference p<0.001 (Table 1, Figure 1).

**TABLE 1:** Body mass differences between groups

	Mean weight (g)	Standard deviation (g)	Minimum (g)	Maximum (g)
WG	44,56	6,22	25	59
EG	42,43	5,62	27	56
WAG	41,40	6,69	24	59

WG=wine group; EG=ethanol group; WAG=water group

### Biochemical analysis

**FIGURE 2:**
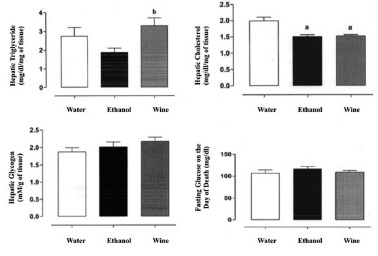
Evaluation of hepatic triglyceride, hepatic cholesterol, hepatic glycogen and fasting glucose on the day of death of the animals

In hepatic triglyceride evaluation the average was higher in WG compared to WAG (18%) and EG (57%), which was 31.6% lower than the control (p<0.01, Figure 2).

In hepatic cholesterol evaluation the average was lower in WG similar to EG (23.6% in wine and 24.5% in ethanol), being statistically significant with p<0.05 (Figure 2).

In hepatic glycogen evaluation the average was higher in WG (16%), not being statistically significant. (Figure 2)

The fasting glucose on the day of animal death the average was higher in EG compared to the other groups, but with no statistically significant difference (p=0.42, Figure 2)

### Fat evaluation

**FIGURE 3:**
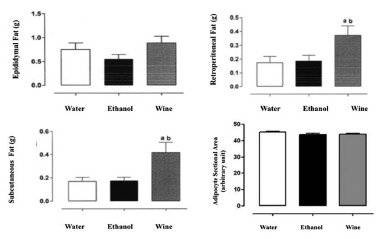
Evaluation of epididymal, retroperitoneal, subcutaneous fat and adipocyte sectional area

In epididymal fat the average weight in grams at was higher in WG (36.5%) and lower in eg (33%, p<0.05, Figure 3).

In retroperitoneal fat the average weight in grams was twice as high in the WG than in the other groups (p=0.026, Figure 3).

In subcutaneous fat evaluation the average weight in grams was 2.3 times higher in the WG than in the other groups, with the difference being statistically significant (p=0.011, Figure 3).

**FIGURE 4:**
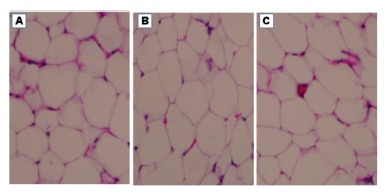
Histological difference between groups: A=WG; B=EG and C=WAG

In adipocyte sectional area evaluation there was no histological difference between the analyzed fats (both representing visceral fat). No significant differences were observed between the groups, that is, there was no adipocyte hypertrophy (Figures 3 and 4)

## DISCUSSION

This study simulated in animals a human-routine habit situation of worldwide trend that is wine consumption in the common diet. Effects on body mass, visceral fat, blood glucose and hepatic steatosis were assessed in clinical use and by measuring the triglyceride, cholesterol and hepatic glycogen in ApoE knockout mice . The ApoE knockout mouse is more suitable for presenting a deficiency in the gene encoding ApoE and for being predisposed to hypercholesterolemia inducing atherosclerosis. This and an already validated model are widely used in trials^3^ ^,^ ^16^ ^,^ ^18^ ^,^ ^20^ ^,^ ^21^ ^,^ ^22^ ^,^ ^25^ ^,^ ^26^.

The choice of wine based on Pinot Noir grape was based in its highest concentrations of resveratrol among all wines (5.13mg/l), which differs from Jonas Lefèvre paper on moderate consumption of red wine that used the Cabernet Sauvignon varietal (4-6 mg/l)^14^.

The determination of the time of trial in four months was due to the fact that these animals have an average life of 12 months and they arrived to the experiment site already with three months of life, therefore, are considered young adults. Based on the trial time, it can be said that the animals underwent to chronic consumption of alcohol stage. 

The body mass gain was higher in WG, which is attributed to its caloric value. Caloric participation is also attributed to ethanol, although alcohol consumption increase can decrease the weight gain^16^.

The hepatic cholesterol accumulation was lower in both EG and WG, suggesting that it is the alcohol that reduces the deposition of cholesterol in the liver of these ApoE knockout animals, which would tend to accumulate cholesterol in the liver. On the other hand, triglycerides had higher accumulation in WG, while decreased in ethanol. Thus, at least for these animals, the ethanol intake was beneficial, while paradoxically the wine was not that good, as it allowed the increase of hepatic triglycerides. Both the glucose and the hepatic glycogen did not differ significantly between groups. The antioxidant property of wine has been widely reported, although many studies have shown that polyphenols may have an unwanted pro-oxidant effect^2^ ^,^ ^4^.

Red wine increases visceral and subcutaneous fat considerably, without affecting the area of ​​the adipocyte, regardless of its alcohol content. It also increases the hepatic triglyceride, in which it also has no regard to the presence of alcohol. Alcohol, either directly or as wine component, decreased hepatic cholesterol accumulation without affecting blood glucose and liver glycogen. It differs from other paper that says that a glass of wine daily inhibits fat accumulation in the liver. This effect arises from the interaction between the alcohol and antioxidant ingredients of the grape. It is possible that the doses help reduce insulin resistance, which contributes to avoid fatty deposition in the liver^4^.

Studies on obesity report reduction of body weight and adiposity with the consumption of resveratrol. In this study, the use of wine, which contains resveratrol, provided greater weight gain to the animals without having adipocyte hypertrophy, which also goes against a research in mice in which the resveratrol did not affect weight gain; however, the highest caloric content of red wine supplement may explain the higher body weight and higher deposits of fat from rats^11^ ^,^ ^27^. 

Fasting glycemia on the day of death of animals was higher in EG. Effect of resveratrol showed a possible hypoglycemic action as well as reduction in the concentrations of lipids and elevation in antioxidant substances, leading to the conclusion that there is hypoglycemic action with the use of wine by the presence of ethanol in its composition^14^.

The use of resveratrol for a long time reduces blood glucose in hyperglycemic conditions; the compound does not affect glucose levels in animals with normal blood glucose. In this study, blood glucose was analyzed only at fasting on the day of death of animals and showed no significant difference.

Recently it was shown that the grape bark extract decreased blood glucose in experimental model of diabetes, which leads to the belief that the use of wine also has the same effect, since the phenolic compounds are present in the grape bark, basic raw material in wine production^21^ ^,^ ^22^.

WG animals, throughout the experiment, were more energetic, observation also shared by another author^19^, although the use has been with resveratrol, polyphenolic component of grape, without the presence of alcohol. In this study, the authors concluded that animals treated with resveratrol showed lower liver damage, reduced risk of developing diabetes and better motor coordination. Thus, the animals that received fatty diet and resveratrol had a longevity and quality of life similar to those who followed a normal balanced diet^26^.

In this study, red wine did not increase the liver glycogen, differing from another which showed that the tannins found in *Vitisvinifera* seeds, and hence in the wine, reduce the level of glucose in the blood, inducing the regeneration of pancreatic cells (epicatechin) inhibiting glucose absorption in the intestine (catechin) and increasing the synthesis of hepatic glycogen (epicatechingallate)^24^.

Weight gain was higher in WG, coming against to this study showing that the use of grape bark extract showed no influence on weight gain or loss. It should be noted that this study did not analyze the influence of the alcohol that is present in the wine. In the same study it was featured that in the animals studied there was a glycemic balance within the normal range without changes in the blood glucose of hypertensive animals.Weight gain had a greater change in WG in relation to the EG. In similar study, ethanol induced rise in total energy intake, as well as in net consumption and palatability, without changing the final weight and weight gain of the experimental animals with equal results compared to the EG with resveratrol^25^.

The effectiveness of red wine consumption on a daily basis in the prevention and treatment of diseases remains controversial , despite a number of studies on resveratrol proving its effectiveness in the prevention and treatment of various diseases. This controversy is mainly due to the presence of alcohol in the composition of the wine that is known to cause dependency^1^ ^,^ ^11^ ^,^ ^14^ ^,^ ^21^.

In spite of the apparent controversial result, the wine worsened lipid metabolism of ApoE knockout mices. The limitation of this study may be exactly the use of these animals, that do not faithfully reproduce what may occur in hypercholesterolemy in humans^20^.

## CONCLUSION

Regular and chronic use of red wine in animals that do not metabolize cholesterol increased hepatic triglyceride and accumulation of visceral and subcutaneous fat. The moderate use of ethanol either alone or associated with wine showed decreased cholesterol, without affecting the mass of adipose tissue.
